# Progerin-Induced Impairment in Wound Healing and Proliferation in Vascular Endothelial Cells

**DOI:** 10.3389/fragi.2022.844885

**Published:** 2022-03-14

**Authors:** Yizhi Jiang, Julie Y. Ji

**Affiliations:** Department of Biomedical Engineering, Indiana University Purdue University Indianapolis, Indianapolis, IN, United States

**Keywords:** progerin, wound healing, shear stress, proliferation, endothelial cells, vascular

## Abstract

Progerin as a mutated isoform of lamin A protein was first known to induce premature atherosclerosis progression in patients with Hutchinson-Gilford progeria syndrome (HGPS), and its role in provoking an inflammatory response in vascular cells and accelerating cell senescence has been investigated recently. However, how progerin triggers endothelial dysfunction that often occurs at the early stage of atherosclerosis in a mechanical environment has not been studied intensively. Here, we generated a stable endothelial cell line that expressed progerin and examined its effects on endothelial wound repair under laminar flow. We found decreased wound healing rate in progerin-expressing ECs under higher shear stress compared with those under low shear. Furthermore, the decreased wound recovery could be due to reduced number of cells at late mitosis, suggesting potential interference by progerin with endothelial proliferation. These findings provided insights into how progerin affects endothelial mechanotransduction and may contribute to the disruption of endothelial integrity in HGPS vasculature, as we continue to examine the mechanistic effect of progerin in shear-induced endothelial functions.

## Introduction

The global prevalence of cardiovascular diseases (CVD) has led to numerous studies investigating the associated lifestyle-related or genetic risk factors. While the mortality rate of CVD has declined in the past few decades, and improvement in cardiovascular health components was observed at all ages, as the aging population steadily increases worldwide, the projected CVD cost would rise sharply by 2035 (Lloyd-Jones et al., 2010; Virani et al., 2020).

The intactness of endothelium is one of the most fundamental indices of vascular healthiness. Endothelial injury or denudation at the local area as well as a delayed or impaired re-endothelialization process could result in the exposure of the internal membrane and vascular smooth muscle cells (VSMCs) to the circulating blood, creating a pro-thrombotic environment that provoked platelet adhesion and the aggregation of inflammatory cells (Shirali et al., 2016; Mastenbroek et al., 2020). Interestingly, the capacity of endothelial cells (ECs) to restore their integrity from injuries can be enhanced by applying normal shear stress (Xia et al., 2012). The correlations between local wall shear stress (WSS) and atherosclerosis revealed by *in vivo* observations and computational modeling studies (Heo et al., 2014; Park et al., 2016) also indicate the beneficial roles of steady laminar flow in providing athero-protective effect *via* regulating EC proliferation and further remodeling process (Gimbrone and Garcia-Cardena, 2013; Roux et al., 2020). On the other hand, disturbed flow found at bifurcations and curvatures posed stress on ECs and resulted in enhanced EC turnover rate at the denuded area (Xu, 2009; Antoniadis et al., 2015).

Lamin A/C, as one of the structural components of the nuclear lamina, not only serves as a mechanically supportive structure but also mediates many cellular events, including cell proliferation by interactions with nuclear membrane proteins and other binding factors (Moiseeva et al., 2011; Vidak et al., 2015). In the vascular system, lamin A/C was also found to be sensitive to mechanical forces and to participate in force-induced cell proliferation, such as its ability to prevent stretch-induced hyperproliferation of VSMC (Han et al., 2015). Genetic mutation in exon 11 on the lamin A/C gene (LMNA) was found to be related to the premature aging process (Simha et al., 2003; Schreiber and Kennedy, 2013). This creates a cryptic splice site responsible for the deletion of 50 amino acids on prelamin A (lamin A precursor) during the post-translational process, resulting in an absence of the recognition site for Zmpste24 (a zinc metallo-endoprotease) to remove the last 15 amino acids at the C-terminus on the precursor. This lack of post-translational modification leads to the generation of a truncated prelamin A called progerin that possesses a permanently farnesylated tail at the C-terminus. Patients carrying that mutation were referred to as having Hutchinson-Gilford progeria syndrome (HGPS). Studies have indicated progerin-induced proliferation impairment in various types of cells and its role in interfering with tissue repair (Wheaton et al., 2017; Hu et al., 2020).

Surprisingly, the McClintock group observed the ubiquitous presence of progerin mRNA transcripts in skin sections derived from healthy donors from all ages, whereas the protein level was elevated with donor’s age (McClintock et al., 2007). Increased misuse of the cryptic splice site on prelamin A was also detected in normal fibroblasts over prolonged *in vitro* culture regardless of donor’s age. Moreover, mechanisms such as telomere shortening, the hyperactivation of p53 tumor suppressor pathway, the extensive damage in DNA repairing, changes in histone methylation, and other age-dependent factors were also found to play a part in both progerin-induced premature aging and normal aging process (Varela et al., 2005; Benson et al., 2010; Holly et al., 2013; Arancio et al., 2014; Kubben et al., 2016; Kubben and Misteli, 2017). In the vascular system, progerin-induced premature aging syndrome also shared many characteristics with physiological aging at cellular and molecular levels (Olive et al., 2010; Hamczyk et al., 2018a). For example, the Ragnauth group found that lamin A precursor was accumulated in late-passage VSMCs or VSMCs derived from elderly donors, which was proposed to be related to the downregulation of the Zmpste24 gene due to its sensitivity toward oxidative stress over passages (Ragnauth et al., 2010). The evidence suggests that progerin may play a part in vascular aging during the normal aging process.

Considering the low number of HGPS individuals worldwide, many progeroid mouse models have been developed to facilitate HGPS research (Hamczyk et al., 2018a), among which the mice that specifically expressed progerin in the vascular system provided valuable information on how the affected vascular cells contribute to cardiovascular dysfunctions. In these models, restrictive progerin expression in VSMCs resulted in arterial structural alternations and contractile impairment (Del Campo et al., 2019; Del Campo et al., 2020), while endothelial progerin expression tended to provoke systematic inflammatory responses, and the endothelium showed defects in response to shear stress (Osmanagic-Myers et al., 2019; Sun et al., 2020). In a progeroid model where only ECs expressed progerin, premature deaths occurred in these mice at the age approaching 25 weeks, probably due to left ventricle diastolic dysfunction. Extensive interstitial fibrosis and adventitial thickening were also reported through histological examination (Osmanagic-Myers et al., 2019).

Besides extracting cells from progeroid mice models, ECs that expressed progerin were also obtained by differentiation from iPSC (induced pluripotent stem cell) derived from fibroblasts of HGPS patients, or by transfection using constructed progerin plasmids. These progerin-expressing ECs presented features of senescence ECs in terms of shortened telomere length and nuclear dysmorphology, as well as cell cycle arrest and reduced proliferation rate (Matrone et al., 2019; Bidault et al., 2020). Attenuated response of gene regulation toward laminar flow (12 dynes/cm^2^) was reported, such as atheroprotective gene KLF2 and the downstream targets of NRF2. On the other hand, the expression of pro-inflammatory genes, including E-selection and VCAM-1, was upregulated. Also, observations of altered NOS regulation and abnormal expression levels of elastin and collagen indicated progerin’s impact on endothelial functions in regulating vascular tone (Atchison et al., 2020).

Although many *in vitro* and *in vivo* studies have been conducted to examine changes in the phenotypes and gene expression profiles of vascular cells expressing progerin, it is not yet known how EC integrity was compromised by progerin to initiate premature atherosclerosis progression in HGPS patients. On the other hand, while flow pattern has been proven to regulate vascular EC functions, few studies were conducted to reveal the relationship between progerin and the local shear stress environments that ECs may be exposed to. In this study, we established stable cell lines of vascular ECs that overexpressed wild-type lamin A or progerin and studied the effects of progerin on the re-endothelialization process under shear stress. Furthermore, the regenerative capabilities of progeria cells at the wound front under different flow patterns were also examined. Our study helps demonstrate how progerin interferes with endothelial wound repair events in a flow environment.

## Materials and Methods

### Cell Culture and Reagents

Bovine aortic endothelial cells (BAEC, pooled donors from Lonza) were grown in Dulbecco’s Modification of Eagle’s Medium (DMEM, Corning) containing 10% heat-inactivated fetal bovine serum (FBS, JR Scientific), 1% L-glutamine, and 2% penicillin–streptomycin (Cellgro). Human embryonic kidney (HEK) cells 293T were grown in DMEM supplemented with 10% FBS and 1% L-glutamine without antibiotics. Cells were maintained in a humidified incubator with 5% CO_2_ at 37°C.

### Stable Transfection

HEK293T cells were utilized to produce the retrovirus carrying either GFP-fused wt-lamin A or progerin gene. Viral production was achieved by the assembly of a retroviral packaging system that contains the packaging plasmid pUMVC (Addgene #8449), the envelope plasmid pCMV-VSV-G (Addgene #8454), and the target plasmid—pBABE-puro-GFP-wt lamin A (Addgene #17662) or pBABE-puro-GFP-progerin (Addgene #17663). Viral supernatants were collected after 48, 72, and 96 h of transfection, and were added on ECs in the presence of polybrene at a concentration of 8 μg/ml. The infection medium was replaced with regular DMEM after 4 h of infection. Drug selection began 48 h after the transduction, and the selection process continued for 10 days until 95% of cells were GFP positive.

### Western Blot

The monolayer of ECs was harvested and lysed by RIPA buffer containing 20 mM Tris-HCl, 150 mM NaCl, 1 mM EDTA, 0.1% SDS, 1% Triton-X, 1 mM DTT, 0.5 mM PMSF, and 150 mM protease inhibitor. Protein concentration was determined by BCA assay and normalized before being loaded on a polyacrylamide gel for electrophoresis. After that, the gel was transferred to a PVDF membrane by the semi-dry blotting method. The membrane was blocked for an hour by 5% BSA or non-fat milk dissolved in 1F0B4 TBST. Primary antibodies included anti-lamin A/C antibody (Cell Signaling, #4777), anti-actin antibody (Sigma, A2066), and anti-GAPDH antibody (Cell Signaling, #2118) as well as corresponding secondary antibodies conjugated with HRP (Bio-rad). Chemiluminescence HRP substrate was used to image the blot. The expression levels of interested bands were normalized by its loading control on ImageJ.

### Nuclear Morphology Quantifications

To characterize nuclei with unusual morphologies, including double nuclei and nuclei with foci and folds, 250–300 cell nuclei were randomly chosen from images acquired from three independent experiments. Results were expressed as the average of the number of these nuclei over the total number of nuclei.

### Wound Healing Assay

Transduced ECs expressing exogenous progerin or wild-type lamin A were seeded on a glass slide at least 24 h before wound formation, and scratches were made by 200-F06Dl pipette tips, with one line parallel to the length of the slide and two lines parallel to the width. Prior to wound healing assay, cells were either kept under static conditions or pre-treated with shear stress for 16 h at 15 dynes/cm^2^, and slides were imaged under the microscope for the next 4 h with 5% CO_2_. Alternatively, the wound was formed before shear application at either low magnitude (2 dynes/cm^2^) or normal magnitude (15 dynes/cm^2^) for 4 h, and slides were imaged as described above. Phase-contrast images were acquired every hour, and the wounded area was quantified at each time point by the wound healing tool on ImageJ. About 10–20 wounded areas were randomly selected for each group for the analysis, and wound length was 974 µm for both horizontal and vertical wounds. Results were presented as recovered area over time, which was calculated by subtracting wounded area at each time point from the initial wounded area at T = 0, and averaged to get the mean value.

### Proliferation Assay

EdU proliferation assay was performed utilizing Click-iT EdU proliferation kit with Alexa Fluor 594 (Invitrogen C10339). Cells were incubated with 10 µM EdU (5-ethynyl-2′-deoxyuridine) in the growth medium for 2 h, after which they were fixed and permeabilized in 3.7% PFA solution and 0.5% Triton X-100, respectively. Fixed samples were then incubated in Click-it reaction cocktail for another 30 min according to the manufacturer’s instruction. Only cells within the first 4 rows at wound edges were included for proliferation analysis. These wounded areas were further categorized by their wound orientation in relation to flow, where the “Horizontal” group denotes the wound edges that were parallel to the shear direction, and “Vertical Downstream” and “Vertical Upstream” denote the wound edges that were oblique to the shear direction and were the downstream and upstream to the flow, respectively. For areas away from wound, 8–15 fields of view at a size of 650 μm^2^ × 487 μm^2^ were randomly chosen for analysis for each group. The proliferation ratio was calculated as the fraction of cells stained positive by Alexa Fluor 594 over the total number of GFP-positive cells. Additionally, to characterize cells at the wounded area that were entering anaphase and telophase after flow application, images were taken at a 5-min interval for 2 h and cells undergoing nuclear expansion and separation were counted. Bi-nucleated cells were also included in the analysis.

### Statistical Analysis

Data were expressed as mean ± SEM. Unpaired two-tailed Student’s *t*-test was performed to compare two groups. To compare more than two groups, ordinary one-way ANOVA was performed and was followed by Tukey multiple comparisons. A *p* value of less than 0.05 was regarded as statistically significant.

## Results

### Confirmation and Characterization of ECs With Exogenous Wild-Type Lamin A or Progerin Expression

**FIGURE 1:**
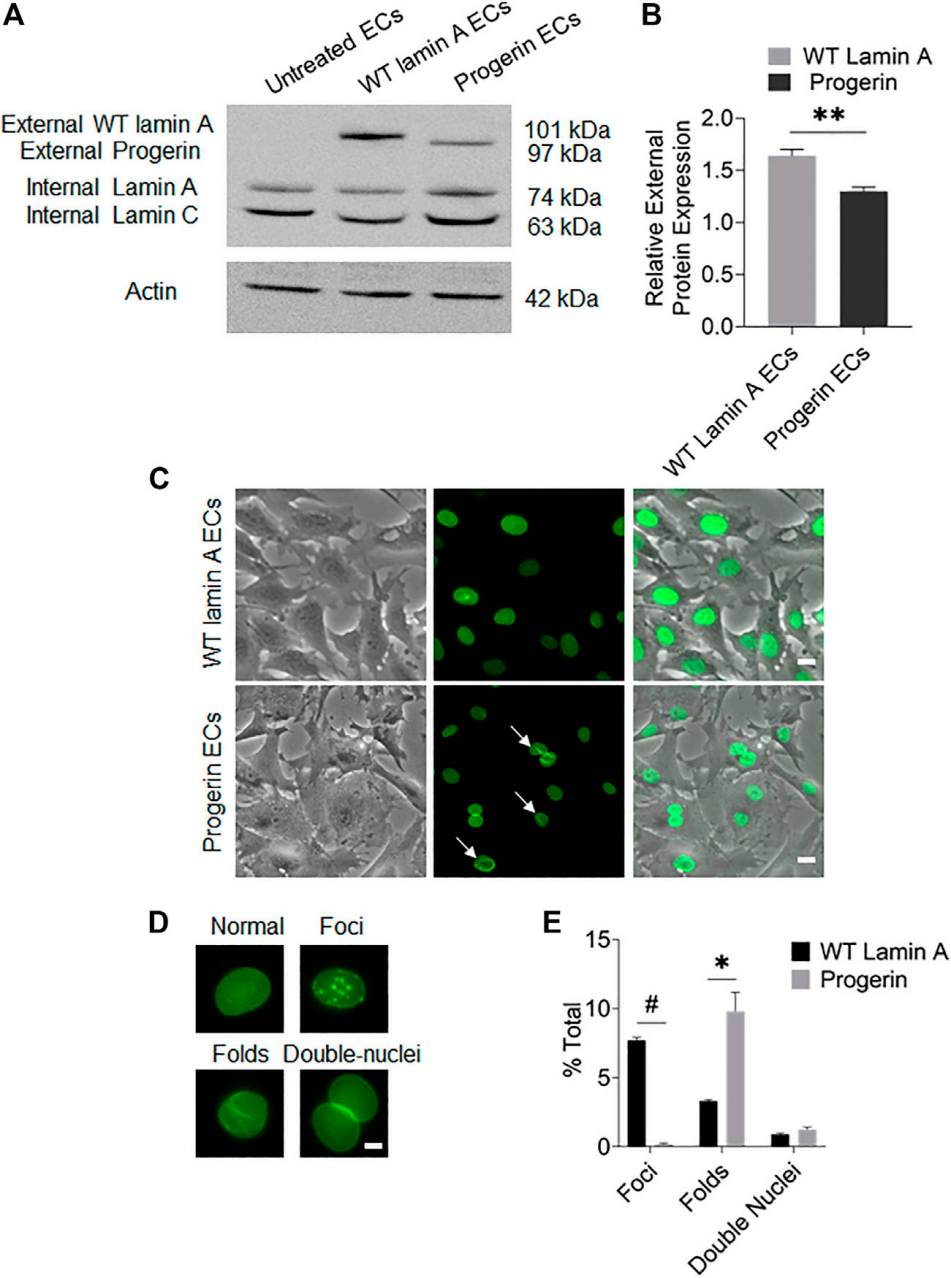
Characterization of targeted protein expression in transduced ECs. **(A)** Western blot showed the presence of endogenous lamin A and exogenous GFP-conjugated proteins in transduced ECs. The sizes of GFP-fused proteins were determined by adding the molecular weights of GFP and the fused protein. **(B)** Quantification of wild-type lamin A and progerin protein expression levels in wild-type lamin A- and progerin-expressing ECs, respectively. **(C)** Representative images of cells showed that the GFP-conjugated proteins were specifically located at nuclei. A substantial population of cells with GFP-progerin expression showed abnormal nuclear shapes (indicated by white arrows). Scale bar: 20 µm. **(D)** Examples of wild-type lamin A-expressing ECs with or without unusual nuclear shapes, including nuclear foci, folds and double nuclei. Scale bar: 5 µm. **(E)** The graph showed the frequency of unusually shaped nuclei in WT lamin A- and progerin-expressing cell population. *p < 0.01, #*p* < 0.001.

The plasmids pBABE-puro-GFP-progerin and pBABE-puro-GFP-wt-lamin were incorporated into host cells using a retroviral packaging system, respectively, to generate cell lines with stable exogenous expressions of these proteins. After selection for single colonies, transduced cells were expanded for stable cells lines. Western blots and fluorescence images were performed to verify the external gene expressions in these cells (Figures 1A, C).

Quantification results of transduced cells with unusual nuclear morphologies (Figure 1E) revealed that a substantial population of progerin-expressing nuclei showed wrinkles and folds, which was also one of the common characteristics of HGPS cells (Denecke et al., 2006; Constantinescu et al., 2010). Wild-type lamin A-expressing cells, on the other hand, showed an increased number of nuclear foci. The Mallampalli group also observed similar trends in their HELA cells that were transiently transfected with GFP-tagged WT lamin A, which was proposed to be related to the overexpression of the external lamin A in cells (Mallampalli et al., 2005). Our quantitative results also indicated a higher expression level of the external protein in WT-lamin A-expressing ECs (Figure 1B). The number of double nuclei was not significantly different between these 2 cell lines.

### Progerin-Expressing ECs Exhibited Differential Wound Healing Responses That Were Dependent on Flow Patterns

**FIGURE 2:**
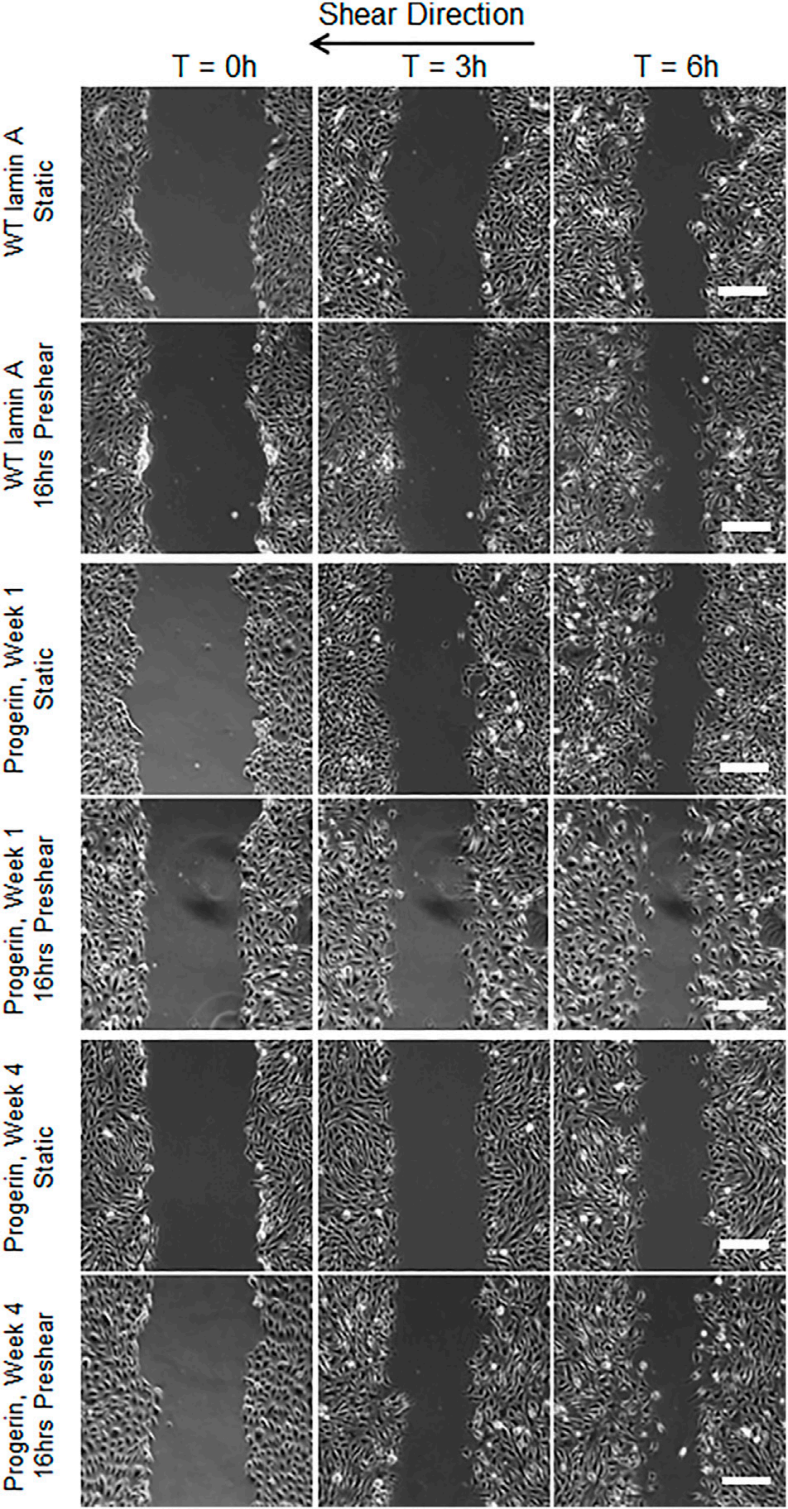
Representative images of wound healing process with or without 16-h-preshearing condition. Wounds were formed following flow application, and images were taken for the next 6 h. Images taken at time points of 0, 3, and 6 h were shown in different columns. Scale bar: 200 µm.

**FIGURE 3:**
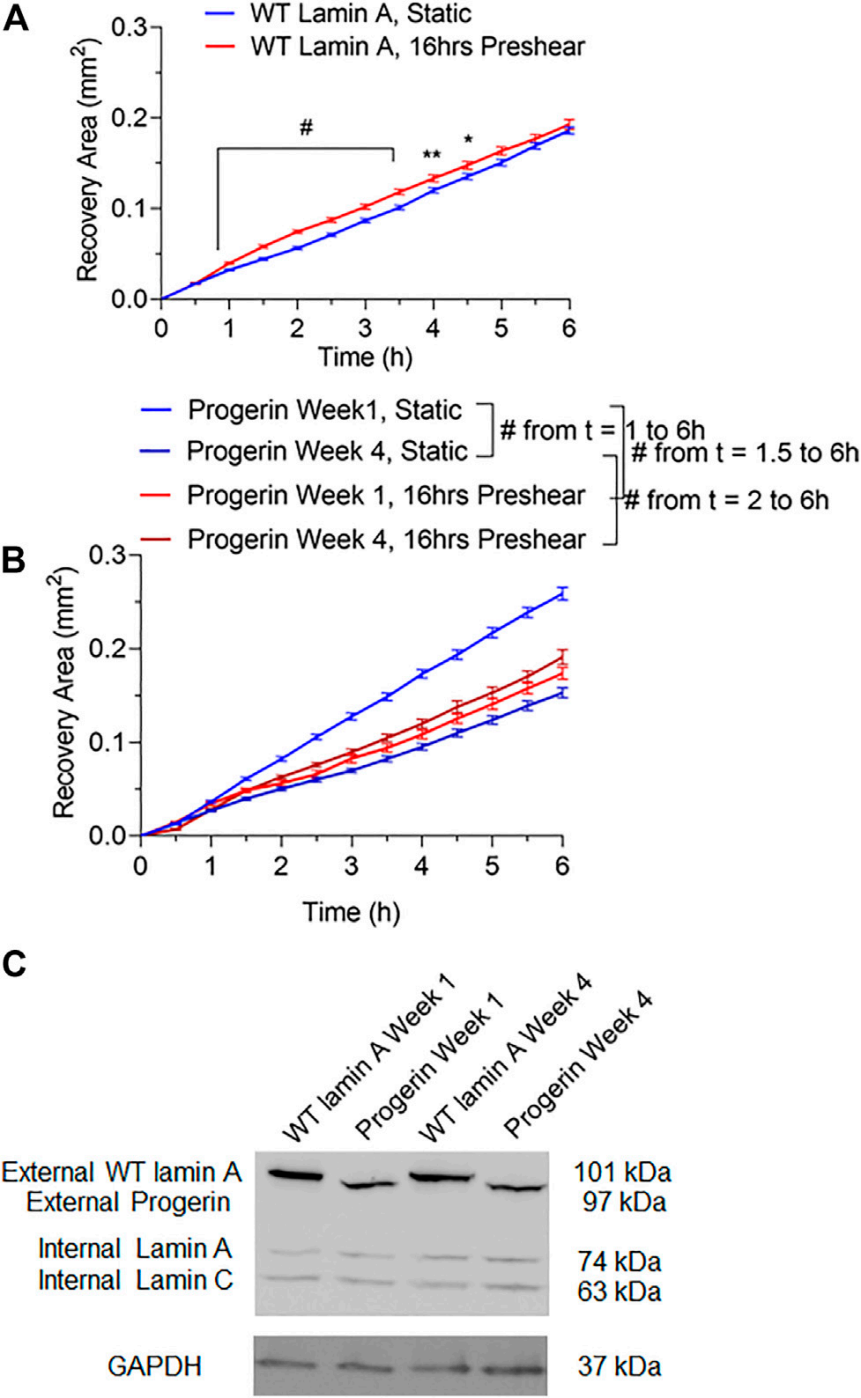
Quantifications of wounded area covered by cells during the 6-h window after wound formation in wild-type lamin A-expressing ECs **(A)** and progerin-expressing ECs **(B)**. Blue lines represent the recovery area of cells that were under static state before wound formation over time, and red lines represent that of cells that underwent 16-h pre-shearing before wounds were created. *p < 0.05, **p < 0.01, #*p* < 0.001. **(C)** Representative Western blot showed external protein expression levels in transduced ECs over serial culture.

Wound repair in WT lamin A- or progerin-expressing endothelial monolayer was investigated by observing wound recovery rate after cells were exposed to acute wounds. First, cells were either pre-treated with normal arterial shear stress of 15 dynes/cm^2^ for 16 h or kept in the incubator before being exposed to acute wound at T = 0 h. Wound recovery for up to 6 h are shown in Figure 2. After recovery areas were quantified, results showed that WT lamin A-expressing ECs with pre-shearing condition showed a faster wound recovery rate than those without flow application (Figure 3A). The trend gradually disappeared after 5 h past wound formation, probably due to the attenuated shearing effects over time. However, progerin-expressing cells exhibited more complex recovery patterns, which were dependent on the length of *in vitro* culture, especially under static state. At the first week upon transfection, progerin-expressing cells exhibited a high recovery rate without flow exposure. However, after 3 weeks of culture, the rate declined substantially (Figure 3B), although the external protein expression levels were not found to be altered over 4 weeks of culture (Figure 3C). This could be due to the reduction in proliferation rate and the associated LAP2 downregulation by progerin over serial culture (Vidak et al., 2015). Interestingly, progerin-expressing cells pretreated with 16-h flow showed similar wound recovery rates regardless of their culture period. To minimize the effects of culture time on transduced cells, only cells cultured within weeks 2 and 3 were used for further examinations, where the proliferation rates of these 2 cell lines were comparable under static state (Figures 5A,B).

**FIGURE 4:**
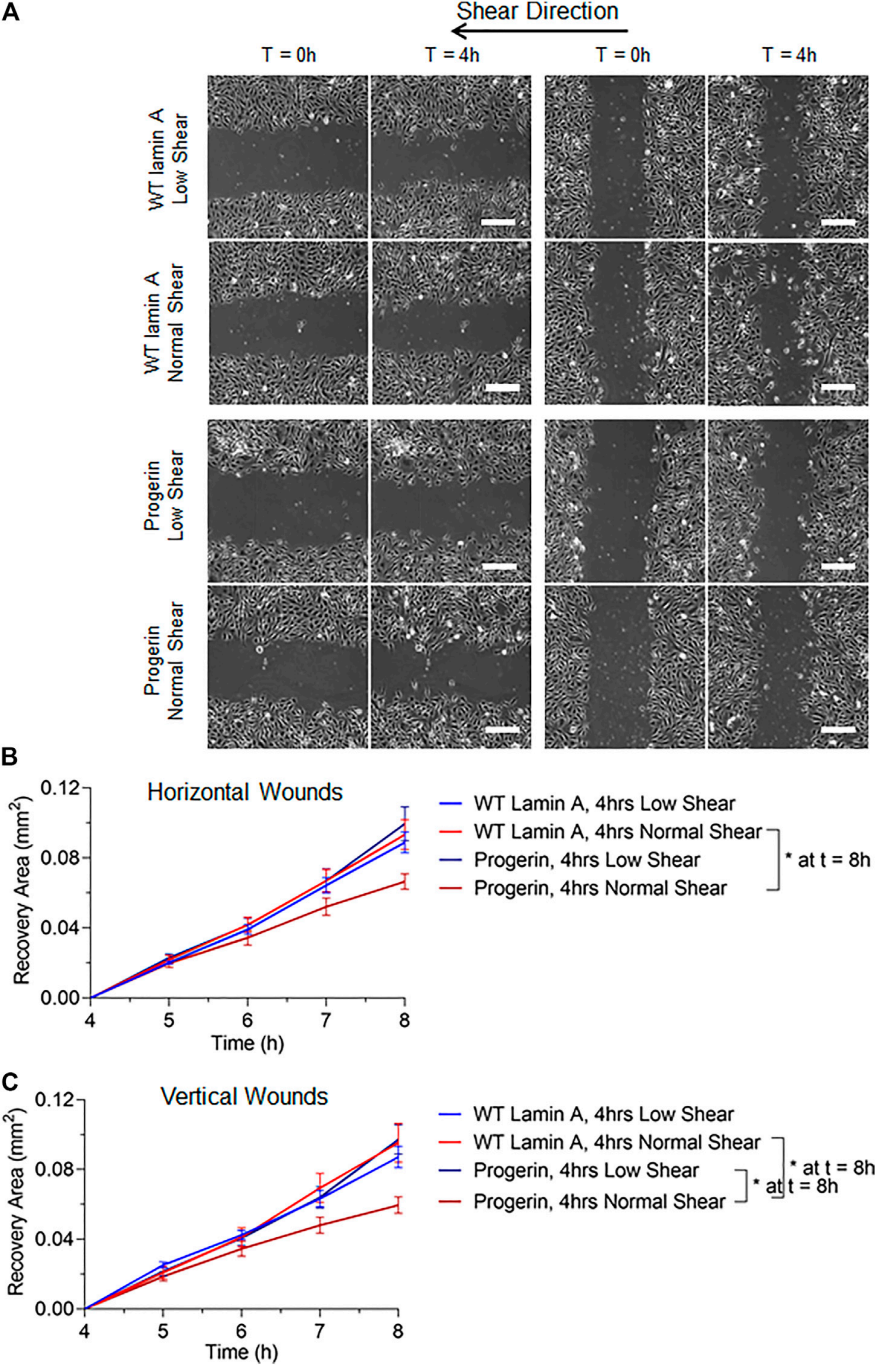
**(A)** Representative images of transduced ECs during the 4-h wound healing window after exposure to flow at low (2 dynes/cm^2^) or normal (15 dynes/cm^2^) magnitude. Scale bar: 200 µm. **(B,C)** Quantifications of wounded area covered by cells after wound formation for wild-type lamin A-expressing ECs and progerin-expressing ECs at horizontal and vertical wounds with shear stress at low or normal magnitude. Blue lines represent the recovery area of cells that were sheared at low magnitude after wound formation, and red lines represent that of cells that were sheared at normal magnitude after wound formation. *p < 0.05.

To better mimic the continual mechanical forces that ECs are exposed to *in vivo*, we also examined the effect of low magnitude shear stress at 2 dynes/cm^2^. In addition, post-shear experiments, where wound was formed before flow applications, were carried out at both low and normal magnitudes for 4 h, to account for wound recovery under flow. Wounded areas were imaged after flow application for the next 4 h, as shown in Figure 4A. Wounds were further categorized as horizontal and vertical wounds in relation to the direction of flow they were exposed. Quantitative results indicated a slowdown in wound recovery rate in progerin-expressing cells after the exposure to normal shear stress regardless of wound orientation (Figures 4B,C). However, these flow patterns did not have a significant effect on the wound recovery rates in WT-lamin A-expressing cells in the 4-h time window. The observed difference in wound recovery event in cells overexpressing progerin may indicate its role in suppressing endothelial wound repair ability and suggest its ability to interfere with endothelial function.

### DNA Replication was Reduced by Normal Shear Stress in Wild-type Lamin A (Compared to Low, Shear Stress), but Not in Progerin-Expressing ECs

**FIGURE 5:**
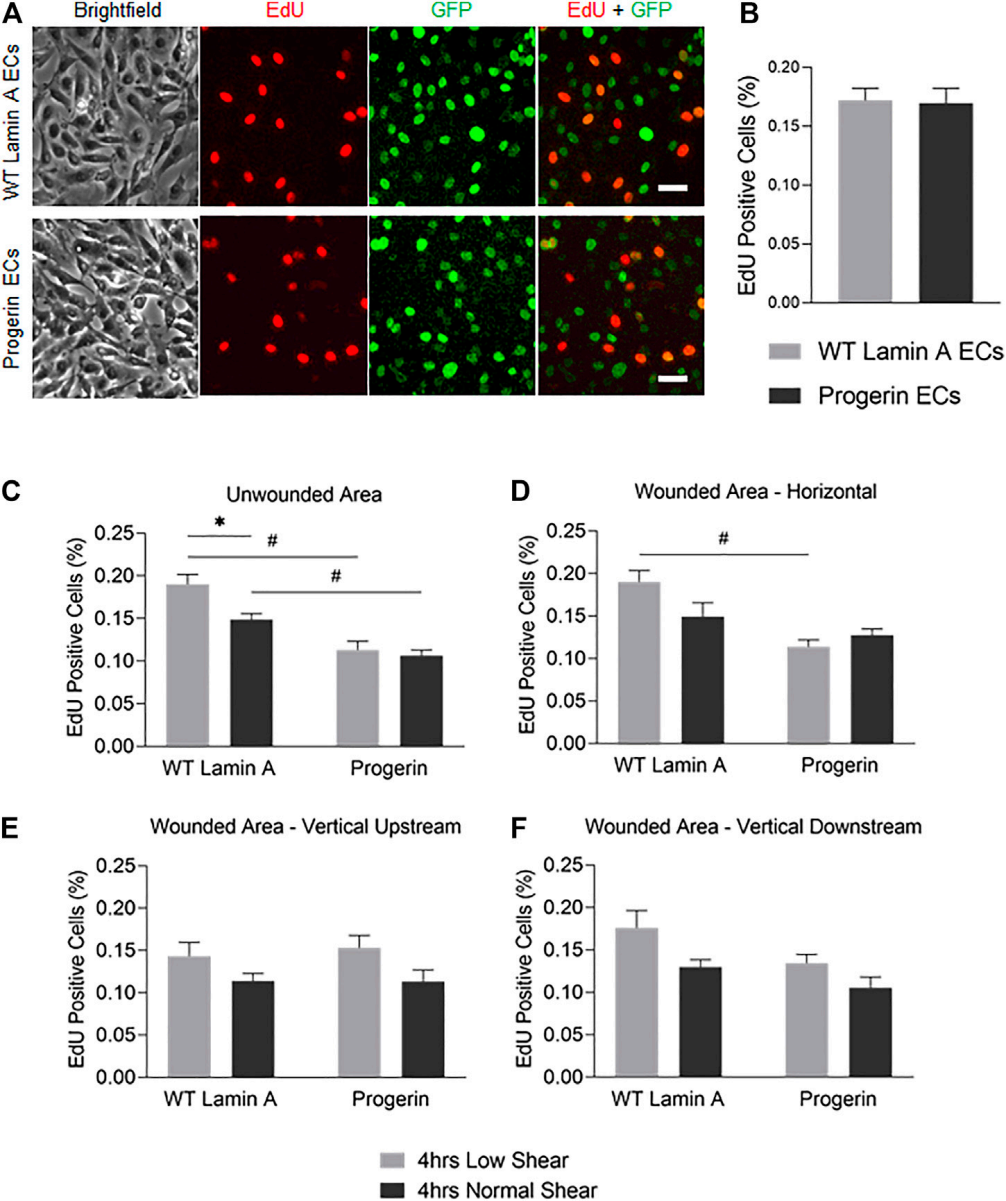
**(A)** Demonstrative images of comparable proliferation rates in wild-type lamin A- and progerin-expressing ECs that were cultured within weeks 2 and 3. Cells were incubated in 10 µM EdU in growth medium for 2 h before fixation. Dyes and channels used: red channel for EdU, green channel for the presence the external proteins fused with GFP. Scale bar: 40 µm. **(B)** Quantification of comparable proliferation rates in transduced ECs that were cultured within weeks 2 and 3. **(C–F)** The percentages of EdU-positive cells at unwounded area **(C)**, horizontal wound edges **(D)**, and vertical wound edges at upstream **(E)** and downstream regions **(F)** as to flow in transduced ECs following flow application. *p < 0.05, #*p* < 0.001.

We next examined if the proliferation potential in progerin-expressing ECs was compromised at the wound edge. Cells were exposed to 4-h laminar flow at low or normal magnitude as described above, after which EdU was added to the growth medium for 2 h before fixation. At regions where endothelial monolayer remained intact, we observed a reduction in the portion of EdU-positive cells in WT-lamin A-expressing cells exposed to normal shear stress, compared to low shear stress. This response is consistent with healthy vascular ECs from literature (Akimoto et al., 2000; Kadohama et al., 2007). On the other hand, cells expressing progerin did not show different DNA replication capacity between low or normal shear stress applied, and the percentages in both conditions were reduced compared to control cells (Figure 5C).

At wound edges, normal shear stress similarly reduced the portion of EdU-positive WT lamin A-expressing cells, compared to low shear, though the difference was more diminished. In contrast, the portion of EdU-positive cells did not change significantly in progerin-expressing cells, at all wound edges (Figures 5D–F). This suggests that wound formation could stimulate DNA synthesis in cells near wound edges, in WT lamin A-expressing ECs under normal shear stress. Progerin-expressing ECs, however, did not show altered portion of cells in S phase, between low and normal shear levels, which would not have contributed to their delayed wound healing response under normal shear (Figure 4).

Overall, the percent of EdU-positive cells show a similar trend between the two shear levels in both WT lamin A and progerin-expressing cells at either vertical wound (Figures 5E,F), suggesting that direction of flow perpendicular to wound did not impact cell proliferation between the cell lines. Also, fractions of EdU-positive cells in both WT lamin A and progerin-expressing cells at the horizontal wound are similar to those observed unwounded area (Figures 5C,D). However, the reduced DNA replication under normal compared to low shear seen in WT lamin A-expressing ECs is absent in progerin-expressing cells, which suggests a sensitivity in cell division toward physiological shear levels that are missing with progerin.

### The Number of Cells Undergoing Late Mitosis at Wound Front Upstream to the Flow Was Reduced in Progerin-Expressing Cells

**FIGURE 6:**
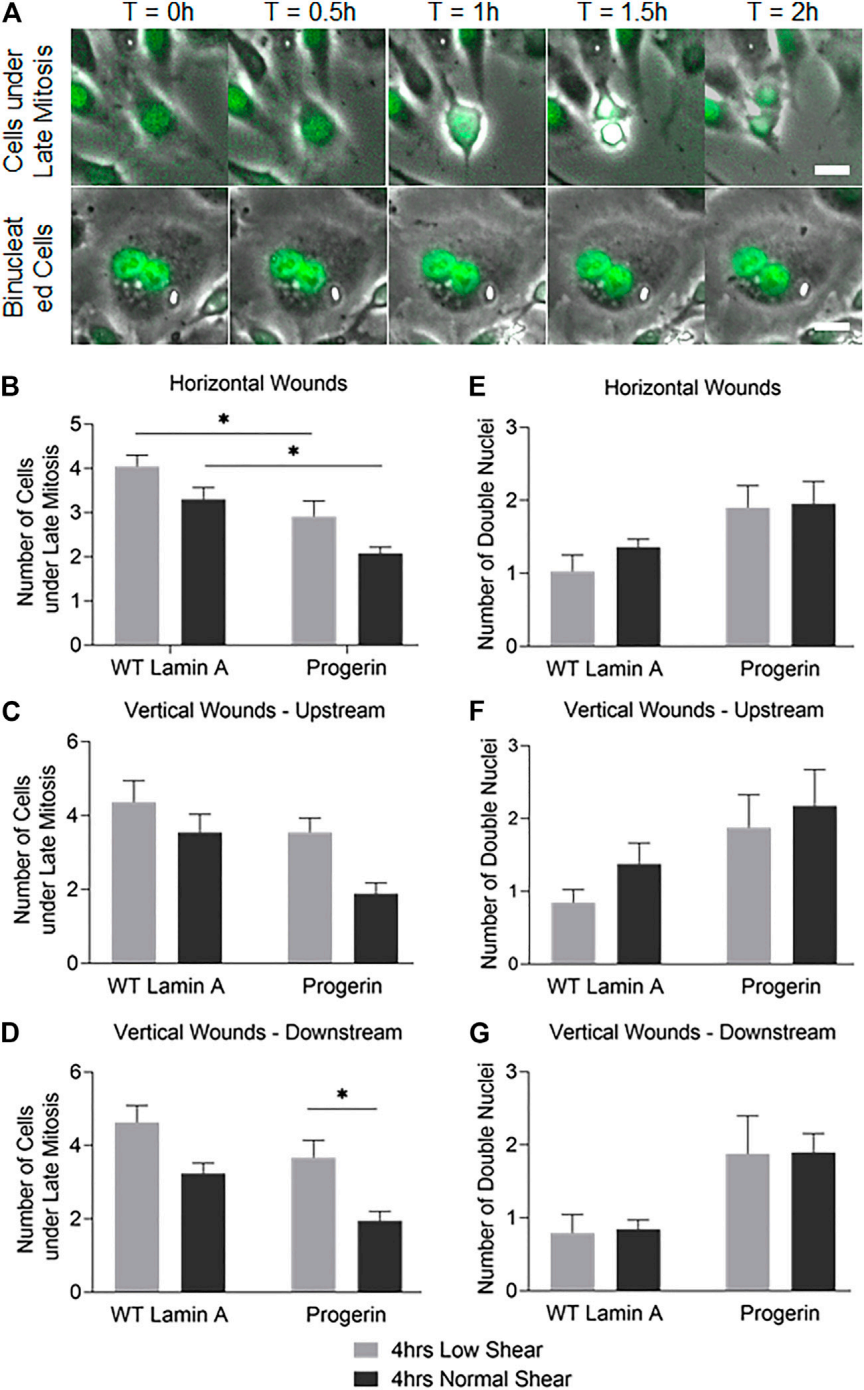
**(A)** Examples of cells under late mitosis that were recognized by nuclear expansion and separation (first row), as well as cells containing double nuclei and were not followed by cytokinesis (second row) during the first 2 h following flow application. Scale bar: 20 µm. **(B–G)** Quantifications of cells that were undergoing late mitosis **(B–D)** or contained double nuclei **(E–G)** within the first 4 rows at horizontal or vertical wound edges (upstream and downstream as to flow) after flow application. *p < 0.05.

To access other cell proliferation activities besides DNA replication, the number of cells that entered anaphase and prophase at the first 4 rows near wound edges were also counted. These were identified by nuclear expansion and separation during the 2-h window after flow was applied (Takemoto et al., 2016), as shown in Figure 6A. Progerin-expressing cells presented an overall reduction in the portion of cells under late mitosis compared to WT lamin A-expressing ECs, especially at horizontal wounds (Figure 6B). Moreover, at wound edges where the wound recovery direction was against the physiological flow, i.e., at vertical downstream edges, the number of cells at anaphase and prophase was also significantly decreased in progerin-expressing ECs (Figure 6D). Within progerin-expressing ECs, the decrease in cells at anaphase and prophase is more pronounced when cells are exposed to the higher, normal shear stress, compared to low shear, especially at vertical downstream edges. On the other hand, the number of binucleated cells in the progerin group is higher than that of the WT lamin A group, regardless of flow condition or shear level (Figures 6E–G). Binucleation appears to be a hallmark of progerin-expressing ECs, and shear-induced binucleation could be a result of cytokinesis failure in progerin-expressing ECs (Nishimura et al., 2016). No significant changes were observed in WT lamin A-expressing cells under different flow conditions, indicating that normal shear stress did not prevent these cells from entering the G2/M phase.

Taken together, our results suggest that the drop in the number of progerin-expressing cells at late mitosis could contribute to delayed wound healing response, especially at normal shear level. The difference in cell cycle observed between WT lamin A- and progerin-expressing ECs in their response toward different shear levels also suggests reduced sensitivity toward mechanical stress in progerin-expressing ECs.

## Discussion

Recent research has highlighted the importance of endothelial and vascular cell dysfunction in HGPS models, as well as progerin-induced mechano-sensitivity in the endothelium (Benedicto et al., 2021). While progerin in endothelial cells has been shown to induce inflammatory responses and impaired mechanotransduction (Osmanagic-Myers et al., 2019). this is a more specific study on how progerin interferes with an endothelial wound recovery. Vascular endothelial injury that may occur *in vivo* following endothelial apoptosis or during post-angioplasty restenosis disrupts endothelial integrity and permeability. Endothelial denudation would expose other vascular cells to the blood flow and trigger a cascade of inflammatory responses (Durand et al., 2004; Curcio et al., 2011). The subsequent healing responses involve endothelial proliferation and migration that occurs under blood flow *in vivo*. Recent studies have revealed the key role of progerin in vascular aging as well as the presence of similar aberrant lamin A isoform in aged people (Minamino and Komuro, 2008; Olive et al., 2010; Ragnauth et al., 2010; Gerhard-Herman et al., 2012). Progerin was also found to impair VSMC proliferation and the metabolism of low-density lipoprotein that involved crosstalk between ECs and VSMCs (Hamczyk et al., 2018b; Goldberg et al., 2018; Hamczyk et al., 2019).

In this paper, we were focusing on the effect that progerin has on endothelial healing response upon denudation under flow, as well as the how cell division and cell cycle during wound recovery are affected in progerin-expressing ECs. We hypothesize that progerin interferes with endothelial integrity under physiological flow and provided evidence of progerin-induced endothelial wound healing delay under normal shear stress (Figure 4). Furthermore, we investigated cell proliferation activities at wound front and wound healing direction relative to flow and found that late mitosis cells at wound edges downstream of flow were most affected by shear stress during the repair event (Figure 6).

Stable cell lines that expressed either wild-type lamin A or progerin protein were generated using a retrovirus packaging system. Wild-type lamin A-expressing cells exhibited nuclei foci that could be due to the overexpression of the external protein, and progerin-expressing cells were characterized as more wrinkles and folds on nuclei. Subsequent wound healing assay revealed delay in endothelial repair in progerin-expressing cells with the application of physiological shear stress, regardless of wound directions (Figure 4). Further data analysis indicated that cell proliferation abilities were compromised at wound front in these ECs, where the number of cells at late mitosis was significantly reduced at vertical wound edges downstream of flow at normal shear stress compared to low shear (Figure 6). These data suggest that endothelial recovery in progerin-expressing ECs was particularly sensitive to physiological shear when it is against the direction of wound recovery.

Additionally, progerin-expressing cells also showed reduced fraction of cells undergoing DNA replication under flow, although the DNA synthesis rate was comparable between wild-type lamin A- and progerin-expressing cells under static state (Figure 5). Normal shear stress reduced number of cells in S phase in WT lamin A-expressing cells, which agrees with previous studies that showed laminar physiological suppressed proliferation as a way to maintain EC quiescent phenotype at athero-protective regions (Chistiakov et al., 2017). The attenuation of fraction of cells in S phase by normal shear is not seen in progerin-expressing cells, which suggests their loss of mechano-sensitivity to shear stress, in regulating the cell cycle.

On the other hand, the number of binucleated cells at the wound front is more prominent in progerin ECs under different patterns of flow (Figure 6). In normal tissues and tumors, cells with double nuclei were also observed and are thought be a result of cytokinesis failure or cell fusion (Nishimura et al., 2016). Although binucleated cells were also present in healthy primary culture, the amount was dramatically increased in HGPS fibroblast and adipocytes, which could be related to abnormal chromosome segregation and accelerated cell senescence (Dominici et al., 2001; Cao et al., 2007; Xiong et al., 2013). However, the underlying mechanisms of binucleated cells remain unknown, and the clinical significance is controversial (Chen et al., 2007).

In summary, we found decreased wound healing rate in progerin-expressing ECs under physiological level of laminar shear stress compared with those under low magnitude shear, and the effect was independent on wound orientations relative to flow direction. Further examination showed that the delayed wound recovery coincided with a reduced number of cells at late mitosis, suggesting potential interference by progerin with delayed cell division. Attenuation of DNA synthesis by normal level of shear stress observed in WT lamin A endothelial cells also disappeared in progerin cells. Both findings support the conclusion that endothelial responses toward athero-protective levels of shear stress were not seen in progerin-expressing ECs, which demonstrates a reduced sensitivity toward mechanical stress in endothelial cells in the presence of progerin. This finding is also in agreement with other recent studies on progerin-expressing endothelium (Osmanagic-Myers et al., 2019).

Research on how progerin affects vascular aging is still ongoing. For example, the exact isoform of prelamin A in elderly people remains unclear. The detection of progerin in cells or tissues derived from normal healthy people has been reported (Scaffidi and Misteli, 2006; McClintock et al., 2007), while the accumulation of the farnesylated prelamin A that retains the 50 amino acids near the carboxyl-terminus was observed in late-passage VSMCs or those from aged donors (Ragnauth et al., 2010). Although the difference in those prelamin A isoforms seemed to be minor in structure, its accumulation mechanisms and downstream effects could be varied.

Moreover, it is still unclear if the toxicity of progerin in HGPS patients was induced by its farnesyl lipid anchor or the 50-amino-acid deletion near the C-terminus. This stems from the purpose of prelamin A processing based on our current understanding. It was found that prelamin A farnesylation in mammalian cells was critical in anchoring lamin A to nuclear lamina during interphase (Lutz et al., 1992; Hennekes and Nigg, 1994). The absence of the farnesylation step on prelamin A also resulted in reduced binding affinity with heterochromatin in HEK293 cells (Lattanzi et al., 2007). However, further studies revealed that the elimination of the farnesylated tail on progerin did not rescue all phenotypes in HGPS cells (Verstraeten et al., 2008). A mouse model that bypassed the modifications and only expressed mature lamin A exhibited normal body weight and survived without developing severe disease phenotypes (Yang et al., 2008; Coffinier et al., 2010).

Current treatment plan for HGPS is still being under development. The first and only drug approved by the US Food and Drug Administration (FDA), Zokinvy (lonafarnib), is a farnesyltransferase inhibitor that detaches progerin from the nuclear membrane. However, farnesyltransferase inhibitors did not rescue all phenotypes in HGPS cells, probably due to the retainment of progerin within nucleoplasm (Verstraeten et al., 2008). Other therapies have been recognized recently, such as isoprenylcysteine carboxyl methyltransferase (ICMT) inhibitor and progerin-lamin A binding inhibitors (Chen et al., 2021; Kang et al., 2021; Macicior et al., 2021; Marcos-Ramiro et al., 2021). More investigations in the functional roles of the deleted 50 amino acids could shed light on alleviating the effect of progerin as well as better HGPS treatment regimens (Lai and Wong, 2020).

We are currently working to continue to examine the mechanistic effect of progerin in shear-induced endothelial functions such as migration, which also plays a key role in the re-endothelialization process. Our findings provided insights into how progerin affects endothelial mechanotransduction in our effort to understand the presence of plaques in HGPS vasculature (Hamczyk et al., 2018a); while further emphasizing the importance of physiological shear in promoting and maintaining endothelial integrity.

## Data Availability

The raw data supporting the conclusions of this article will be made available by the authors, without undue reservation.
